# Characterization of Bovine Foamy Virus Gag Late Assembly Domain Motifs and Their Role in Recruiting ESCRT for Budding

**DOI:** 10.3390/v14030522

**Published:** 2022-03-03

**Authors:** Zhaohuan Wang, Rui Li, Chenxi Liu, Wentao Qiao, Juan Tan

**Affiliations:** Key Laboratory of Molecular Microbiology and Technology, Ministry of Education, College of Life Sciences, Nankai University, Tianjin 300071, China; 1120190482@mail.nankai.edu.cn (Z.W.); 2120191012@mail.nankai.edu.cn (R.L.); 2120201045@mail.nankai.edu.cn (C.L.); wentaoqiao@nankai.edu.cn (W.Q.)

**Keywords:** bovine foamy virus, L domain, ESCRT, virus-like particles

## Abstract

A large number of retroviruses, such as human immunodeficiency virus (HIV) and prototype foamy virus (PFV), recruit the endosomal sorting complex required for transport (ESCRT) through the late domain (L domain) on the Gag structural protein for virus budding. However, little is known about the molecular mechanism of bovine foamy virus (BFV) budding. In the present study, we report that BFV recruits ESCRT for budding through the L domain of Gag. Specifically, knockdown of *VPS4* (encoding vacuolar protein sorting 4), *ALIX* (encoding ALG-2-interacting protein X), and *TSG101* (encoding tumor susceptibility 101) indicated that BFV uses ESCRT for budding. Mutational analysis of BFV Gag (BGag) showed that, in contrast to the classical L domain motifs, BGag contains two motifs, P_56_LPI and Y_103_GPL, with L domain functions. In addition, the two L domains are necessary for the cytoplasmic localization of BGag, which is important for effective budding. Furthermore, we demonstrated that the functional site of Alix is V498 in the V domain and the functional site of Tsg101 is N69 in the UBC-like domain for BFV budding. Taken together, these results demonstrate that BFV recruits ESCRT for budding through the PLPI and YGPL L domain motifs in BGag.

## 1. Introduction

Foamy viruses (FVs), or spumaretroviruses, are a group of *Retroviridae* that use a different replication pathway from orthoretroviruses [1,2]. These differences include the following: infectious FV particles contain double-stranded DNA, indicating that reverse transcription occurs in the virus particles before a new round of infection; instead of producing Gag–Pol fusion proteins, PFV Pol proteins are translated from spliced mRNA [3]; an internally functionally active second transcription unit for the expression of unstructured genes is present in the FV genome [4]; with respect to budding, FV Gag proteins lack a membrane-targeting signal, and therefore cannot produce cell-free Gag-only virus-like particles, thus FV is dependent on the capsid–glycoprotein interaction to provide a membrane-targeting function for Gag. However, studies have shown that if FV Gag proteins are fused with Fyn or Lck myristoylation to enable Gag to target the membrane, particles can be released independently of Env. These observations indicate that FV Gag contains all the other structural motifs necessary for capsid assembly and budding [5,6]. FVs infect humans and other mammals, including simians, equines, bovines, and felines [7,8,9,10]. The FV replication strategy represents a link between the *Retroviridae* and the *Hepadnaviridae*, which makes FVs interesting research subjects.

The release of viral particles from infected cells is one of the last steps in the retroviral replication cycle, in which budding from the cellular membrane or organelle membrane is a critical step in a highly coordinated process, usually aided by a number of cellular factors [11]. Retroviruses are able to recruit endosomal sorting complex required for transport (ESCRT) components through interactions mediated by one or more specific motifs on their capsid precursor protein to complete budding [12]. These specific motifs play an important role in late events in the intracellular virus life cycle; therefore, the motifs on retroviral Gag that interact with ESCRT components were referred to as “late assembly” or L domains [13,14]. To date, three typical L domain sequences have been characterized. The P(T/S)AP L domain motif was first confirmed in the human immunodeficiency virus type 1 (HIV-1) Gag p6 domain [15]; the YPXL L domain motif was first found in the equine infectious anemia virus (EIAV) Gag p9 domain [16]; and the PPXY L domain motif was originally identified in the Rous sarcoma virus (RSV) Gag p2b cleavage product [17,18]. Recently, the LXXL L domain motif was found in HIV and EIAV, which overlaps with the YPXL motif [19]. Typically, a retroviral Gag that promotes budding by recruiting ESCRT contains one or more L domain motifs [11,20].

The ESCRT components are utilized in the cell to bud cargo-enriched vesicles into the lumen of multivesicular bodies (MVBs) and mediate membrane scission events to release the vesicles. The ESCRT machinery also plays a role in the membrane scission events in the final steps of cell division [12,21]. These processes are topologically identical to virus budding. Different types of L domain motifs interact with different components of ESCRT. The P(T/S)AP motif interacts with tumor susceptibility 101 (Tsg101), a component of the ESCRT I complex, to mediate the budding of viruses containing this L domain motif, such as HIV-1 [22,23]. The YPXL motif recruits ESCRT through interaction with ALG-2-interacting protein X (AIP-1/Alix) and then mediates virus budding [24]. The recruitment of ESCRT by the PPXY motif is related to its binding with the WW domains of a subset of NEDD4 E3 ubiquitin protein ligases (NEDD4)-like homologous to E6AP C-terminus (HECT) ubiquitin ligases; however, the specific mechanism remains unclear [25,26].

Compared with orthoretroviruses, there have been few studies on the motifs associated with budding of FV Gag. In the case of FVs, it has been confirmed that PSAP is the L domain motif in prototype foamy virus (PFV) Gag, which can recruit ESCRT through an interaction with Tsg101 to mediate PFV budding [27]. Surprisingly, the Gag proteins of all non-primate FVs, including bovine foamy virus (BFV), do not have the P(S/T)AP motif. Therefore, whether the budding of these non-primate FVs is ESCRT-dependent, and whether they contain undiscovered motifs with L domain function requires investigation.

In the present study, we aimed to characterize whether the BFV Gag protein (BGag) has L domain functional motifs, to analyze their role in the particle budding process, and determine their relationship with ESCRT components.

## 2. Materials and Methods

### 2.1. Cell Culture and Transfection

Human embryonic kidney (HEK293T), MDBK, and HeLa cells were cultivated in Dulbecco’s modified Eagle’s medium (Gibco, Thermo Fisher Scientific, Waltham, MA, USA), supplemented with 10% fetal bovine serum (FBS) (Gibco), 50 µg/mL streptomycin, and 50 U/mL penicillin at 37 °C in a 5% CO_2_ atmosphere.

For transfection, cells were seeded at 70–80% confluence in either 6-well plates, 12-well plates, or 10 cm dishes. Twenty-four hours later, the required plasmids were transfected into the cells. The polyethylenimine (PEI, Polysciences, Warrington, PA, USA) used in the experiment was added at a DNA:PEI (µg:µg) ratio of 1:4, according to the manufacturer’s protocol. After ten minutes, the mixture was added to the corresponding cells. Small interfering RNA (siRNA) transfection was performed using lipofectamine 3000 (Life Technologies, Grand Island, NY, USA) according to the manufacturer’s instructions.

### 2.2. Plasmid Constructs

Human *ALIX* and human *TSG101* cDNAs were cloned into vector pCMV-3HA (Clontech, Mountain View, CA, USA). Different site mutants (pCMV-3HA-Alix V498D, pCMV-3HA-Tsg101 Y63A, and N69P) were generated using site-directed mutagenesis (Toyobo, Osaka, Japan) according to the manufacturer’s recommendations.

The primers sequence used to construct the pCMV-3HA-Alix V498D mutant were: forward primer—5′-GGAACCAACTTCAGAACAGATTTAGATAAAGCTGTGCAG-3; reverse primer—5′-CTGCACAGCTTTATCTAAATCTGTTCTGAAGTTGGTTCC-3′. The primers sequence used to construct the pCMV-3HA-Tsg101 Y63A mutant were: forward primer—5′-GGAACAATCCCTGTGCCTGCTAGAGGTAATACATAC-3; reverse primer—5′-GTATGTATTACCTCTAGCAGGCACAGGGATTGTTCC-3′. The primers sequence used to construct the pCMV-3HA-Tsg101 N69P mutant were: forward primer—5′-GAGGTAATACATACCCTATTCCAATATGCCTATGG-3; reverse primer—5′-CCATAGGCATATTGGAATAGGGTATGTATTACCTC-3′.

The coding sequence of BFV Env was inserted into pCMV-3HA to construct the pCMV-3HA-BEnv plasmid, and the coding sequence of BFV Gag was cloned into pCE-puro-3×FLAG to construct the pCE-puro-3×FLAG-BGag plasmid. Mutations were generated by designing specific mutation primers and using site-directed polymerase chain reaction (PCR) (Toyobo, Osaka, Japan).

All mutant plasmids were verified by sequencing before use (Genewiz, Beijing, China).

### 2.3. siRNA Construction

*VPS4* (encoding vacuolar protein sorting 4) expresses two transcripts, *VPS4A* and *VPS4B*. The *VPS4*-specific siRNA (5′-GGAUGUCCCUGGAGAUAAAtt-3′), which targeted both transcripts, and a negative control siRNA (NC), were purchased from GenePharma (Shanghai, China).

The siRNA (5′-GAACAAAUGCAGUGAUAUA-3′) specifically targeting position 2063 to 2081 bp of *ALIX* and an NC siRNA were purchased from GenePharma.

The siRNA (5′-CCUCCAGUCUUCUCUCGUC-3′) specifically targeting position 414 to 432 bp of *TSG101* and an NC siRNA were purchased from GenePharma.

### 2.4. Quantitative Real-Time Reverse Transcription PCR (qRT-PCR)

Total RNA was extracted using the TRIzol Reagent (Invitrogen, Waltham, MA, USA) according to the manufacturer’s protocol. The extracted RNA was reverse transcribed into cDNA, and the cDNA was used as a template to perform quantitative real-time PCR on the StepOnePlus Real-Time PCR System (Applied Biosystems, Foster City, CA, USA), using FastStart Universal SYBR Green PCR Master Mix (Roche, Basel, Switzerland). *GAPDH* (encoding glyceraldehyde-3-phosphate dehydrogenase) was used as an internal control.

qPCR was performed using the following conditions: 94 °C for 3 min for 1 cycle; 94 °C for 30 s, 60 °C for 30 s, and 72 °C for 30 s, for 40 cycles. The sequences of the primers used in the experiment were: VPS4A-up: 5′-GTGATGGAGAAGCCCAACATAC-3′; VPS4A-low: 5′-CAAGTGTGGGAATTTGATTGGC-3′; VPS4B-up: 5′-CGACCAAATGTGAAATGGAGTGA-3′; VPS4B-low: 5′-TCCAGGCGGCCCAAATAATAG-3′.

After determining the specificity of amplification by melting curve analysis, the relative expression of the target mRNA was calculate using the 2^−ΔΔCT^ method.

### 2.5. Purification of BFV Virus-like Particles (VLPs)

At 48 h after transfection, the cell culture supernatant containing BFV VLPs (including the VLPs released by Env alone and the VLPs formed by Env and Gag) were filtered through a 0.45 µm filter, and then 1 mL of 20% sucrose buffer (weight/volume) was added to the centrifuge tube, and then the prepared VLPs were added to the upper layer of sucrose. Ultracentrifugation (Optima LE-80K, Beckman Coulter, Indianapolis, IN, USA) at 4 °C, 35,000 rpm was performed for 2 h, and the invisible pellet was resuspended in 40 µL of loading buffer containing 2% SDS, and it was stored at −20 °C before immunoblotting.

### 2.6. Western Blotting Analysis

Transfected cells were disrupted using lysis buffer (150 Mm NaCl, 50 Mm Tris, 2 Mm EDTA, 3% Glycerol, 1% NP-40) on ice for 30 min, and then protein loading buffer was added containing 2% SDS. Proteins were separated by sodium dodecyl sulfate polyacrylamide gel electrophoresis (SDS-PAGE). Thereafter, the proteins were transferred from the gel to a polyvinylidene difluoride (PVDF) membrane (GE Healthcare, Cincinnati, OH, USA) by electroblotting at 95 V at 4 °C for 90 min. The PVDF membrane was then incubated in phosphate-buffered saline (PBS) containing 5% nonfat milk for 45 min at room temperature, followed by incubation with the primary antibodies for 1.5 h. The blot was the incubated with species-specific, peroxidase-conjugated secondary antibodies and then with a chemiluminescent substrate reagent for visualization. The immunoreactive proteins were detected using chemiluminescence (Merck Millipore, Darmstadt, Germany).

### 2.7. Immunofluorescent Assay

HeLa cells were seeded in 12-well plates with coverslips at the bottom, and transfected with corresponding plasmids. Two days later, the cells were treated with 500 µL of fixative (PBS containing 4% formaldehyde) for 10 min, and then treated with PBS containing 0.1% Triton X-100 for 10 min to perforate the cell membrane surface. Next, 500 µL of PBS containing 5% non-fat milk and 5% BSA were added to the plates and incubated at room temperature for 2 h or at 4 °C overnights for blocking. Cells on coverslips were incubated with the primary antibody (1:250 dilution of the mouse anti-Flag antibody) at room temperature for 2 h or at 4 °C overnight, washed 3 times using PBS, and incubated for 40 min with fluorochrome-conjugated secondary antibodies in the dark. The coverslips were washed 3 times with PBS for 5 min each time, and then 500 µL of 4′,6-diamidino-2-phenylindole (DAPI) was added to the plates and incubated for 10 min in the dark. The coverslips were washed four times with PBS, fixed on the slides using glycerol, and air-dried at room temperature in the dark. The samples were stored at 4 °C in the dark for long-term storage. Images were captured under a confocal fluorescence microscope (Leica TCS SP5, Wetzlar, Germany).

### 2.8. Co-Immunoprecipitation

HEK293T cells were seeded in a 10 cm dishes and transfected with the corresponding plasmids after 24 h. After 2 days, the cells were disrupted using lysis buffer containing 50 mM Tris, 150 mM NaCl, 2 mM EDTA, 3% Glycerol, 1% NP-40, and EDTA-free protease inhibitor cocktail tablets for 1 h on ice, and then centrifuged at 10,000× *g* for 10 min at 4 °C. The sample were incubated with protein A agarose beads (cat. no. 16-125, Millipore, Boston, MA, USA) for 3 h at 4 °C with rotation. The samples were centrifuged at 4 °C, 10,000× *g* for 1 min to remove the supernatant, and the immunoprecipitated components in the pellet were washed with lysis buffer 6 times, and the supernatant was removed after centrifugation at 4 °C, 10,000× *g*. After the last washing, the supernatant was removed using a 1 mL syringe and only the immunoprecipitated components were retained. An equal volume 2 × loading buffer containing 2% SDS was added to the samples for 20 min at 100 °C and then the samples were subjected to Western blotting.

### 2.9. Separation of Cell Nucleus and Cell Cytoplasm

The cells (dish) were collected and washed 3 times with ice-cold PBS and centrifuged at 4 °C, 3000 rpm for 5 min each time. After the final PBS wash, the cells were resuspended in 500 µL Buffer A (protease inhibitor, 1 M HEPES, 2 M KCl, 1 M MgCl_2_, 1 M DTT), and then incubated on ice for 15 min. The cells were passed through the needle of a 1 mL syringe (26 G) 5–10 times and then left on ice for 15 min. The cells were centrifuged at 2800 rpm for 5 min at 4 °C: The supernatant contained the cytoplasm and the pellet contained the cell nuclei. The pellet was resuspended in 1 mL of solution I (0.25 M sucrose, 10 mM MgCl_2_, protease inhibitor), and the resuspended pellet was layered over 3 mL of solution II (0.35 M sucrose, 0.5 mM MgCl_2_, protease inhibitor), and then centrifuged at 1430× *g* for 5 min at 4 °C. This step resulted in a cleaner nuclear pellet. The nuclear pellet was resuspended in 100 µL Buffer A. The samples were stored at −80 °C, before being subjected to Western blotting.

### 2.10. Antibodies

Antibodies used for protein analysis were as follows: monoclonal mouse anti-HA (1:5000; cat. no. H3663, Sigma-Aldrich, St. Louis, MO, USA), monoclonal mouse anti-Flag (1:5000; cat. no. F1804, Sigma-Aldrich), monoclonal rabbit anti-Alix-N-terminus (1:2000; cat. no. ab186429, Abcam, Cambridge, MA, USA), polyclonal rabbit anti-Vps4A/B (1:2000; cat. no. 17673-1-AP, Proteintech, Chicago, IL, USA), monoclonal mouse anti-Tsg101 (1:1000; cat. no. ab83, Abcam, Cambridge), Alexa Fluor-488-conjugated goat anti-mouse IgG (1:5,00; cat. no. A-11001, Invitrogen, Carlsbad, CA, USA), monoclonal mouse anti-GAPDH (1:5000; cat. no. sc-47724, Santa Cruz Biotechnology, Dallas, TX, USA), monoclonal mouse anti-Tubulin (1:5000; cat. no. sc-32293, Santa Cruz), polyclonal rabbit anti-Histone H3 (1:2000; cat. no. ab1791, Abcam), horseradish peroxidase (HRP)-conjugated goat anti-mouse IgG (1:5000; cat. no. sc-2005, Santa Cruz), and HRP-conjugated goat anti-rabbit IgG (1:5000; cat. no. sc-2004, Santa Cruz).

### 2.11. Statistical Analysis

To quantify the levels of released VLPs, the amount of Gag in the VLPs was normalized against the amount of intracellular Gag, which were first normalized against the GAPDH loading control. In the Western blotting experiments, the corresponding immunoreactive protein band intensities were determined using Image J [28,29].

All data were expressed as the mean ± standard deviation (SD) of the results of three independent experiments, and each experiment was conducted three times. Comparisons between two groups were performed using Student’s t-test with GraphPad Prism version 8.0 (GraphPad software Inc., San Diego, CA, USA). When the P value was less than 0.05, the difference was considered statistically significant. The P values in the figures are expressed as * *p* < 0.05, ** *p* < 0.001, *** *p* < 0.0001, and not significant (ns) (for *p* > 0.05).

## 3. Results

### 3.1. Alix and Tsg101 Are Necessary for the Budding of BFV VLPs

Recent studies have shown that the ESCRT pathway is the main escape route of enveloped viruses, which is closely related to the L domains on viral structural proteins [30,31]. To investigate whether budding of BFV also depends on ESCRT, we examined the effect of knockdown of ESCRT components on BFV VLP formation. The bovine Alix and Vps4 sequences cannot be found in NCBI and studies have suggested that BFV zoonotic infection might be possible [32]; therefore, we attempted to conduct experiments based on human ESCRT proteins. We also compared the sequence similarity between human and bovine ESCRT proteins, and found that the human Tsg101 (NP_006283.1) protein and the bovine Tsg101 (NP_001091464.1) protein had a high sequence similarity of 97.44%. In addition, we used antibodies recognizing human ESCRT protein to detect the corresponding proteins in MDBK cells. As shown in Figure S1A, Alix, Vps4, or Tsg101 in MDBK cells could be detected by rabbit anti-homo Alix, rabbit anti-homo Vps4 or mouse anti-homo Tsg101 antibodies, suggesting high similarity of ESCRT proteins between humans and cows. For the above reasons, we conducted the follow-up experiments based on human derived Alix, Tsg101, or Vps4.

**Figure 1:**
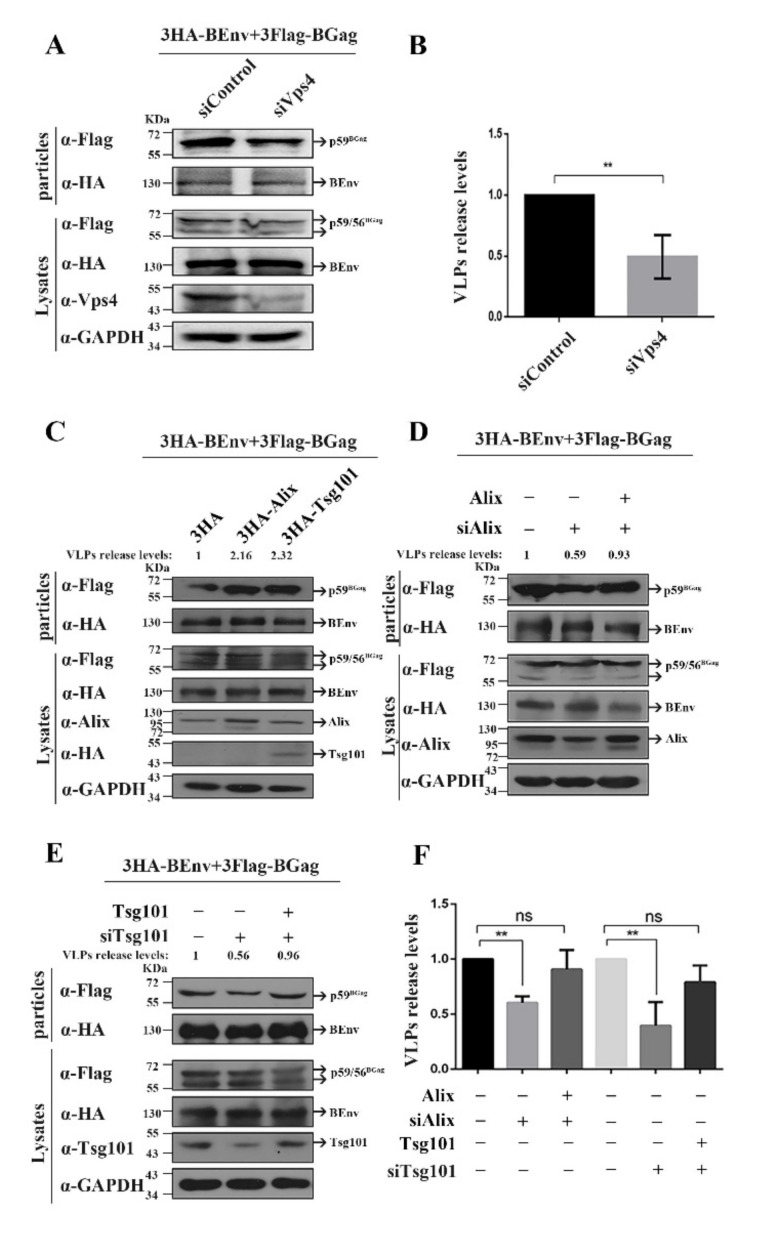
Effect of Vps4, Tsg101, or Alix proteins on particle budding. (**A**) HEK293T (4 × 10^6^) cells were transfected with si*VPS4* or siControl 6 h before being transfected with 3HA-BEnv or 3Flag-BGag (BFV Gag protein), and then cultured for 24 h. The cell culture supernatants were filtered through a 0.45 µm filter and purified by ultracentrifugation. Transfected cells were lysed using lysis buffer. Levels of proteins in cells and supernatants were measured using Western blot. (**B**,**F**) To quantify the levels of released VLPs, the amount of BGag in VLPs was normalized against the amount of intracellular BGag, which was first normalized against the GAPDH loading control. Mean values and the standard deviation of particle-associated BGag protein corrected for intracellular expression levels (*n* = 3) are shown. The data are the averages of three independent experiments. Compared with the siControl: ** *p* < 0.01. (**C**) HEK293T (4 × 10^6^) cells were transfected with 3HA-BEnv, 3FlagBGag, and either the 3HA, 3HA-Alix, or 3HA-Tsg101 vector constructs and harvested at day 2 post-transfection. The cell culture supernatants were filtered through a 0.45 µm filter and purified by ultracentrifugation. Transfected cells were disrupted using lysis buffer. Levels of proteins in cells and supernatants were measured using Western blotting. (**D**) HEK293T (4 × 10^6^) cells were transfected with siAlix or siControl 6 h before transfected with 3HA-BEnv, 3Flag-BGag, and either the pCMV-3HA or pCMV-3HA-Alix vector constructs, and then cultured for 24 h. The cell culture supernatants were filtered through a 0.45 µm filter and purified by ultracentrifugation. Levels of proteins in cells and supernatants were measured using Western blotting. (**E**) HEK293T (4 × 10^6^) cells were transfected with siTsg101 or siControl 6 h before transfected with 3HA-BEnv, 3Flag-BGag, and either the pCMV-3HA or pCMV-3HA-Tsg101 vector constructs, and then cultured for 24 h. The cell culture supernatants were filtered through a 0.45 µm filter and purified by ultracentrifugation. The levels of released VLPs were quantified according to the method described in the statistical analysis section.

Vps4, as an ATPase, is necessary for ESCRT-dependent virus budding [33,34]; therefore, we examined the budding of BFV VLPs in *VPS4* (siVPS4 can target both *VPS4A* and *VPS4B* transcripts) knockdown HEK293T cells. In this study, we co-transfected the pCMV-3HA-BEnv plasmid with the pCE-puro-3×FLAG-BGag plasmid to detect the production of VLPs. Additionally, the secretion of non-infectious subviral particles (Env-only SVPs) is also a feature of the spumaretroviruses-specific budding strategy, and this situation might only be a byproduct of the unique Env-dependent egress strategy of infectious FV particles [5]. Therefore, in this study, under the condition of co-transfection of pCMV-3HA-BEnv with pCE-puro-3×Flag-BGag plasmid, the Env protein detected in the supernatant contains two components: including Env-only SVPs and Gag-Env VLPs, thus the total amount of Env in supernatant did not change with changing Gag levels. As shown in Figure 1A,B, *VPS4* knockdown reduced the amount of Gag in BFV VLPs to about 49.5%. These results indicated that BFV budding is ESCRT-dependent. 

Previous studies have shown that Alix and Tsg101 are key factors of the ESCRT pathway, and different types of L domains recruit ESCRT complexes by interacting with them. To determine whether BFV recruits ESCRT through Alix and Tsg101, we examined the effects of *ALIX* and *TSG101* knockdown on BFV VLP release. As shown in Figure 1D–F, *ALIX* and *TSG101* knockdown inhibited the budding of BFV VLPs, and the corresponding inhibition could be compensated for by transfection of exogenous *ALIX* or *TSG101*. Correspondingly, overexpression of *ALIX* or *TSG101* promoted the budding of BFV VLPs (Figure 1C). Taken together, these results demonstrated that Alix and Tsg101 are necessary for BFV to recruit ESCRT complexes.

### 3.2. Identification of L Domain Sequences on the BGag Protein

**Figure 2:**
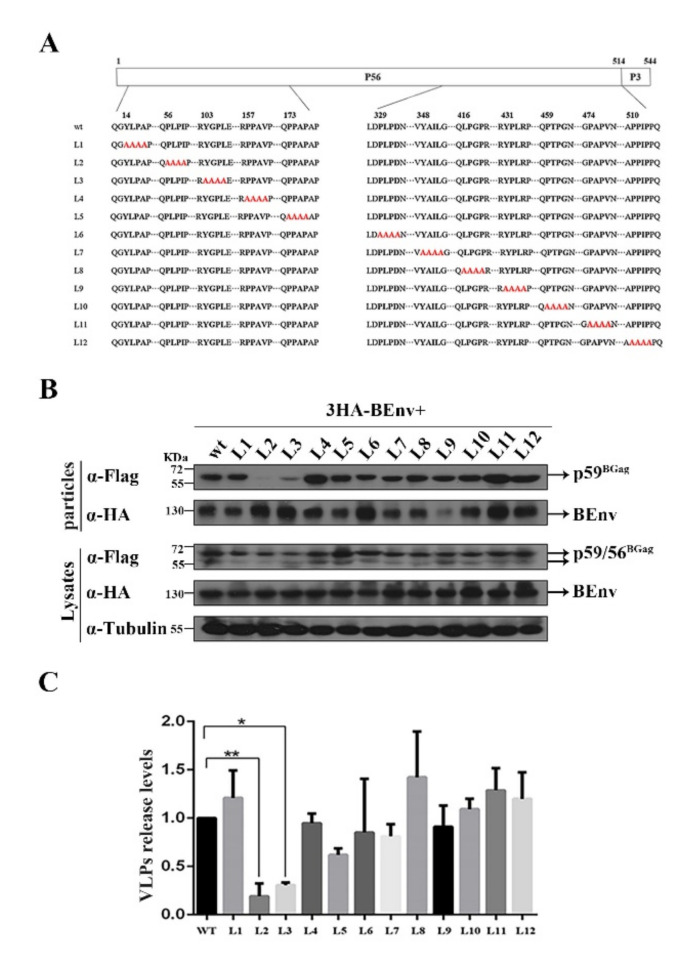
Schematic illustration of the BGag mutants and the impact of each mutant on BFV VLP budding. (**A**) Schematic organization of the BFV Gag protein (BGag) precursor protein and processing products p56 and p3. Below, the sequences of the indicated specific regions of wild-type and mutant BGag proteins are shown. Putative L domain sequence motifs are highlighted in bold, and amino acids altered in the mutant constructs are marked by red letters. (**B**) HEK293T (4 × 10^6^) cells were transfected with 3HA-BEnv and either the wild-type or domain-mutated (3Flag-BGag L1–L12) vector constructs and harvested at day 2 post-transfection. The cell culture supernatants were filtered through a 0.45 µm filter and purified by ultracentrifugation. Transfected cells were disrupted using lysis buffer. Levels of proteins in cells and supernatants were measured using Western blotting. (**C**) Quantification of BFV virus-like particle (VLP) budding. Mean values and standard deviation of particle-associated BGag protein corrected for intracellular expression levels (*n* = 3) are shown. To quantify the VLPs release levels, the amount of BGag in VLPs was normalized against the amount of intracellular BGag, which were first normalized against the GAPDH loading control. The data are the averages of three independent experiments. Compared with the wild type (wt): * *p* < 0.05, ** *p* < 0.01. “·” stands for omission.

BFV depends on the ESCRT pathway for budding; therefore, we hypothesized that there would be L domain motifs in BGag. Three classes of L domain sequences, based on the peptide sequences P(T/S)AP, PPXY, and (L)YPX_n_L, have been described in retroviruses. Analysis of the amino acid sequence of BGag identified no classical L domain motifs. To determine the possible L domain (s) in BGag, we mutated twelve motifs (L1–L12) in BGag that closely resembled the three classes of L domain sequences (Figure 2A).

**Figure 3:**
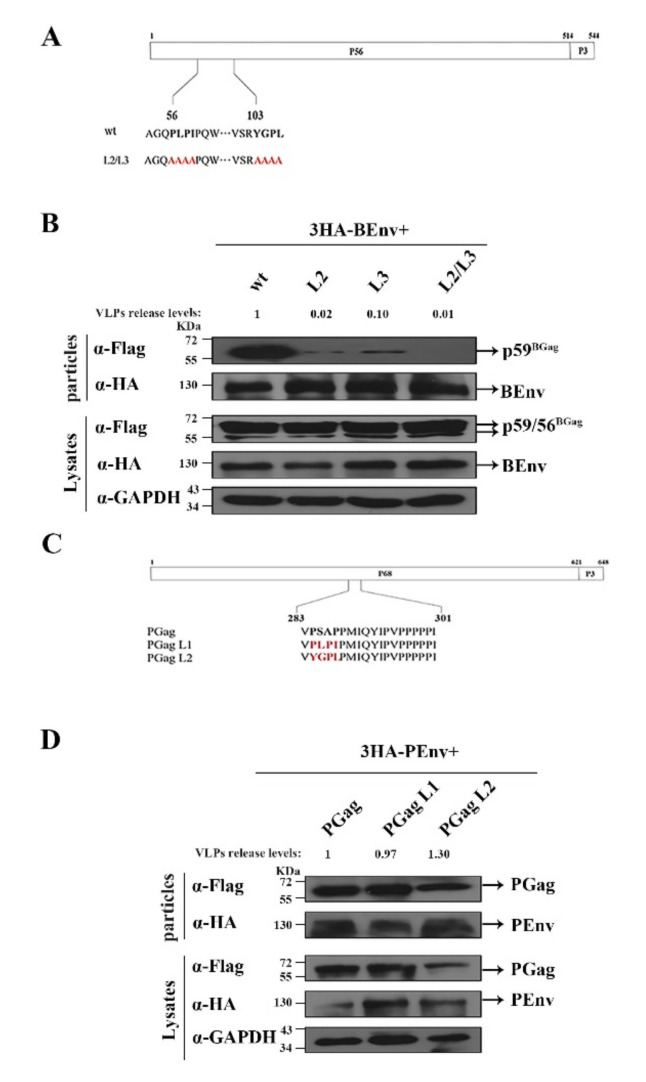
PLPI and YGPL are the two L domains in BGag. (**A**) Schematic organization of the BFV Gag protein (BGag) precursor protein and processing products p56 and p3. Below, the sequences of the indicated specific regions of wild-type and mutant BGag protein are shown. The two L domain sequence motifs are highlighted in bold, and amino acids altered in the mutant constructs are marked by red letters. (**B**) Representative Western blotting analysis of HEK293T cell lysates (Lysates) and virus-like particles (VLPs) purified by ultracentrifugation through 20% sucrose using monoclonal anti-Flag (α-BGag) and anti-HA (α-BEnv)-specific antisera. To quantify the levels of released VLPs, the amount of BGag in VLPs was normalized against the amount of intracellular BGag, which were first normalized against the GAPDH loading control. (**C**) Replacement of PFV L domain motif with BFV L domain motifs. Schematic organization of the PFV Gag protein (PGag). The PGag L domain sequence motif is highlighted in bold, and amino acids altered in the mutant constructs are marked by red letters. (**D**) HEK293T (4 × 10^6^) cells were transfected with 3HA-PEnv and either the wild-type or domain-mutated PGag vector constructs and harvested at day 2 post-transfection. The cell culture supernatants were filtered through a 0.45 µm filter and purified by ultracentrifugation. Transfected cells were disrupted using lysis buffer. Levels of Gag and Env in the supernatant and cell lysate were measured using Western blotting. To quantify the levels of released VLPs, the amount of PGag in VLPs was normalized against the amount of intracellular PGag, which were first normalized against the GAPDH loading control.

To identify the effect of different Gag mutants on BFV VLP budding, a BFV Env (BEnv) expression plasmid and plasmids expressing different BGag proteins were co-transfected into HEK293T cells. At 2 days post-transfection, levels of BGag and BEnv in the cells and supernatants were measured (including Env-only SVPs and Gag-Env VLPs). As shown in Figure 2B,C, mutations in the PLPI (L2) and YGPL (L3) motifs resulted in a reduction in the fraction of BGag that was present in VLPs. The combination mutant (L2/L3) resulted in a more obvious reduction in BGag levels in VLPs, and BGag was almost undetectable in the supernatants (Figure 3A,B).

Previous studies have shown that L domains of different viruses are functionally interchangeable [35,36,37]. To further prove that PLPI and YGPL motifs are L domains, we replaced the L domains in PFV Gag (PGag) with PLPI and YGPL (Figure 3C), and then a PFV Env (PEnv) expression plasmids and plasmids expressing different PGags were co-transfected into HEK293T cells. At 48 h, the levels of PGag and PEnv in cells and supernatants were measured. As shown in Figure 3D, the levels of VLPs released from different PGag mutants were not lower than those of the wild-type, although the expression levels of the PGag L2 mutant were lower than those of the wild-type. This result indicated that PLPI and YGPL can replace the L domain function of PFV to maintain the normal budding of PFV VLPs. We also examined the effect of L domain mutation on budding of BFV VLPs in the MDBK cell lines. As shown in Figure S1B, mutation of the L2/L3 domains inhibited the budding of BFV VLPs (VLPs release levels were 57% compared with that of the of WT). This was consistent with the results of the experiments conducted in HEK293T cells (Figure 3B).

Taken together, these data indicated that the PLPI and YGPL motifs in BGag contain the L domain activity for BFV particle budding, which is also consistent with the result that BFV budding is ESCRT-dependent (Figure 1).

### 3.3. The Two L Domains of BGag Are Required for Its Cytoplasmic Localization

**Figure 4:**
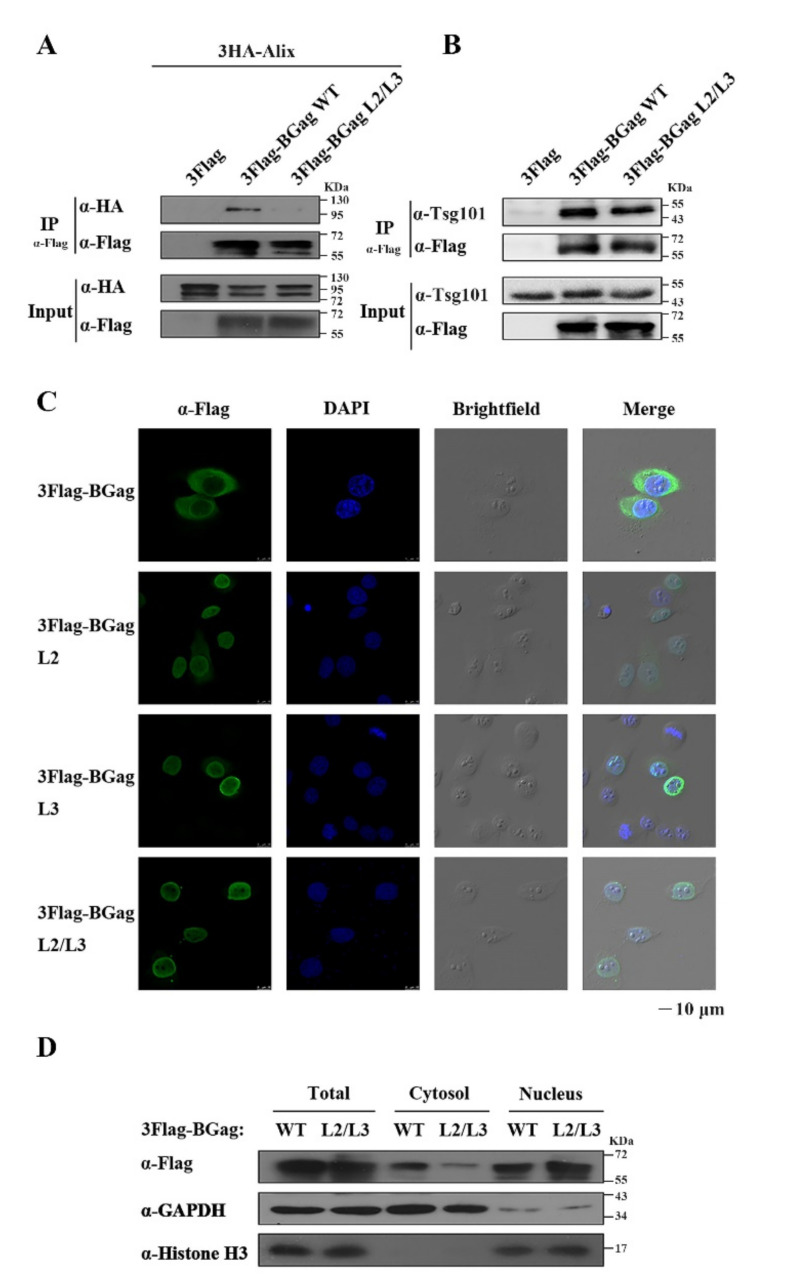
The localization of wild-type or L domain motifs-mutated BGag. (**A**) Immunoprecipitation with Flag antibody of HEK293T (4 × 10^6^) cells co-transfected with eukaryotic expression plasmids encoding 3Flag-Gag or 3Flag-Gag L2/L3 and 3HA-Alix. (**B**) Immunoprecipitation with mouse anti-Flag antibody of HEK293T (4 × 10^6^) cells transfected with eukaryotic expression plasmids encoding 3Flag-Gag or 3Flag-Gag L2/L3. The co-immunoprecipitated Tsg101 was detected using mouse anti-Tsg101 antibody, and the co-immunoprecipitated BGag protein was detected using mouse anti-Flag antibody. Tsg101 and BGag protein levels in the input were also detected using Tsg101 and Flag antibodies, respectively. (**C**) HeLa cells were transfected with 3Flag-BGag, 3Flag-BGag L2, 3Flag-BGag L3, or 3Flag-BGag L2/L3 (BFV Gag protein (BGag)). An indirect immunofluorescence assay (IFA) was used to localize wild-type or L domain motifs-mutated BGag (with mouse anti-Flag antibody and fluorescein isothiocyanate (FITC)-conjugated goat anti-mouse secondary antibody). Green represented the BGag protein, blue represented the nucleus, and brightfield showed the cell morphology. Scale bar = 10 μm. (**D**) HEK293T cells were transfected with either 3Flag-Gag or 3Flag-Gag L2/L3. After 2 days, the cells were collected, and the nucleus and cytoplasmic components were separated. The BGag protein levels in the two components were determined using Western blotting. Histone 3 (H3) and glyceraldehyde-3-phosphate dehydrogenase (GAPDH) were used as nuclear and cytoplasmic markers, respectively.

The nascent FV Gag precursor was reported to transiently traffic through the nucleus during particle assembly [38]. The subsequent efficient transport of FV Gag from the nucleus to the cytoplasm is necessary for the completion of budding, which undoubtedly depends on the interaction between Gag and the intracellular transport system. The L domains are related to the ESCRT pathway; therefore, we confirmed the interaction between BGag (wild-type or L domains mutated) and Alix or Tsg101 using immunoprecipitation. The results showed that the interaction between BGag and Alix was weakened significantly after the L domains were mutated compared with that of wild-type BGag (Figure 4A). There was a slight weakening of the interaction between BGag and Tsg101 after the L domains were mutated (Figure 4B).

Therefore, we examined the effect of the two L domains on the subcellular localization of BGag using immunofluorescence detection. As shown in Figure 4C, the wild-type BGag was mainly localized in the cytoplasm, while the BGag L2/L3 mutant was almost exclusively localized in the nucleus. The nuclear localization levels of the two mutants, BGag L2 and BGag L3, also increased markedly. We further examined the subcellular localization of wild-type BGag and its L2/L3 mutant by performing a nuclear and cytoplasmic fractionation assay. The distribution of L2/L3 mutated BGag in the cytosol was about 1/3 that of the wild-type BGag, and its distribution in the nucleus was about 1.7 times that of the wild-type BGag (Figure 4D and Figure S2A). 

These results suggested that the two L domains are important for the cytoplasmic localization of BGag during budding.

### 3.4. V498 in the V Domain of Alix Is Crucial for BFV VLP Budding

**Figure 5:**
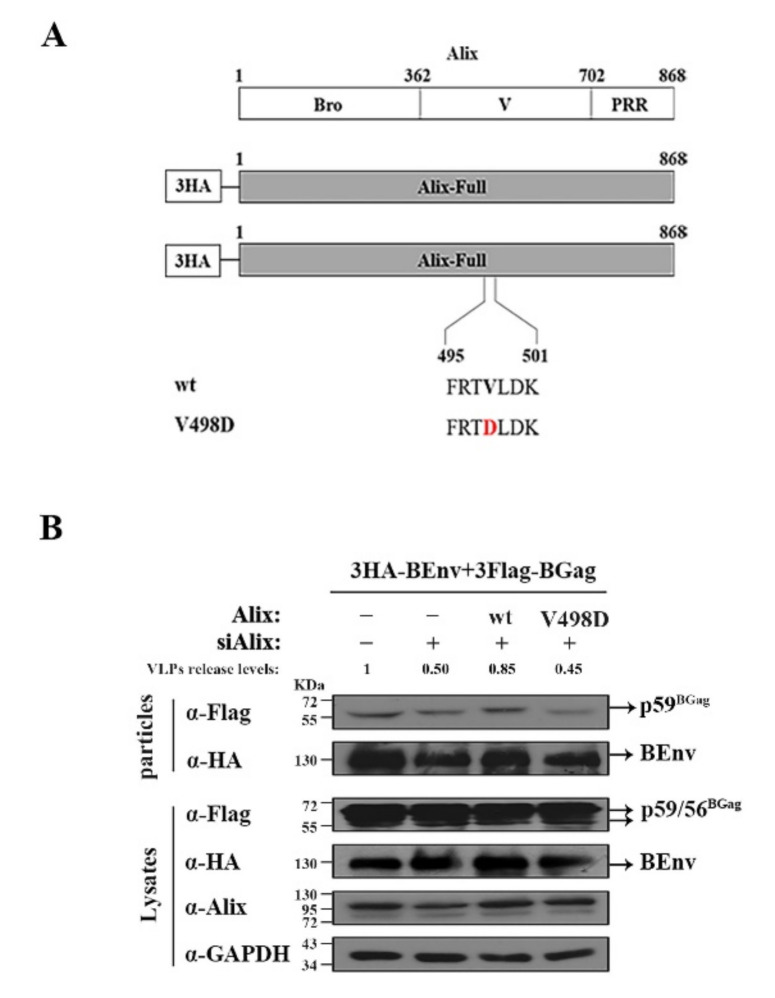
V498 in the V domain of Alix is crucial for BFV VLP budding. (**A**) Various forms of Alix (including full-length and the V498D mutant) were cloned into pCMV-3HA. The sequences of the indicated specific regions of wild-type and mutant Alix protein are shown. Amino acid altered in the mutant constructs is marked by red letters. (**B**) HEK293T (4 × 10^6^) cells were transfected with siControl or siAlix 6 h before transfection with 3HA-BEnv, 3Flag-BGag (BFV Gag protein (BGag)), and either the pCMV-3HA or various forms of Alix vector constructs were then cultured for 24 h. The cell culture supernatants were filtered through a 0.45 µm filter and purified by ultracentrifugation. Transfected cells were disrupted using lysis buffer. Levels of proteins in cells and supernatants were measured using Western blotting.

Alix is necessary for BFV to recruit ESCRT; therefore, we further identified the functional amino acid site(s) of Alix. Previous studies have shown that the V498 amino acid inside the V domain of Alix is very important for its biological function [39,40]. To determine the effect of V498 in Alix on BFV budding, a plasmid with the V498D mutation was constructed (Figure 5A). HEK293T cells were transfected with siControl or siAlix, and then the corresponding cells were transfected with the same amount of pCMV-3HA, pCMV-3HA-Alix, or pCMV-3HA-Alix V498D DNA constructs. The results showed that knockdown of *ALIX* inhibited the budding of VLP, and full-length Alix (the third lane, Figure 5B) could compensate for this inhibition, while Alix with the V498D mutation (the fourth lane, Figure 5B) could not compensate for this inhibition (Figure 5B). Overall, these results indicate that the V498 in the V domain is the functional site through which Alix assists BFV budding.

### 3.5. N69 in the UBC-like Domain of Tsg101 Is Crucial for BFV VLP Budding

**Figure 6:**
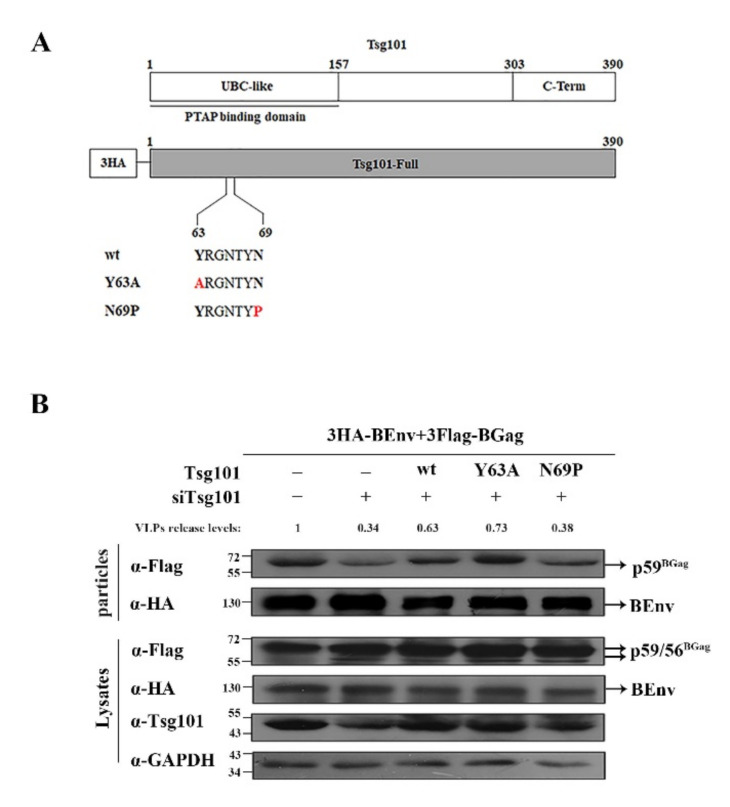
N69 in the UBC-like domain of Tsg101 is crucial for BFV VLP budding. (**A**) Various forms of Tsg101 (including full length, and Y63A and N69P mutants) were cloned into pCMV-3HA. The sequences of the indicated specific regions of wild-type and mutant Tsg101 protein are shown. Amino acids altered in the mutant constructs are marked by red letters. (**B**) HEK293T (4 × 10^6^) cells were transfected with siControl or siTsg101 6 h before transfection with 3HA-BEnv, 3Flag-BGag (BFV Gag protein (BGag)), and either the pCMV-3HA or various forms of Tsg101 vector constructs were then cultured for 24 h. The cell culture supernatants were filtered through a 0.45 µm filter and purified by ultracentrifugation. Transfected cells were disrupted using lysis buffer. Levels of proteins in cells and supernatants were measured using Western blotting.

The Tsg101 UBC-like domain (1–157 aa) was shown previously to be the binding domain of the PTAP L domain [41]. To identify the functional site(s) of Tsg101 that assist BFV budding, as shown in Figure 6A, we constructed two Tsg101 mutants, Y63A and N69P [42]. HEK293T cells were transfected with siControl or siTsg101, and then the corresponding cells were transfected with the same amount of pCMV-3HA or various forms of Tsg101 (including full-length Y63A and N69P). The results showed that Y63A (the fourth lane, Figure 6B) could compensate for the inhibition of budding induced by *TSG101* knockdown, akin to the effect of transfection with the plasmid expressing full-length Tsg101 (the third lane, Figure 6B), while N69P (the fifth lane, Figure 6B) had no compensatory ability (Figure 6B). These results identified amino acid N69 in the UBC-like domain as the functional site of Tsg101 that assists BFV budding.

## 4. Discussion

Successful egress from infected cells is a prerequisite for virus spread within the host. In this study, we reported that BFV also buds through recruitment of the ESCRT pathway, similar to most retroviruses. We also identified that PLPI and YGPL are the two L domain motifs in BGag. In particular, mutation experiments showed that the two L domains of BGag were necessary for effective budding and also affected the distribution of Gag between the nucleus and cytoplasm.

Our results clearly demonstrated that BFV requires late components of the Vps machinery for particle egress. SiRNA-mediated knockdown of *VPS4A* and *VPS4B* (encoding AAA ATPases) inhibited BFV particle release. The majority of retroviruses express one or two L domains in a specific protein, and the use of two distinct L domains have equivalent functions in virus release [5]. The HIV-1 Gag protein contains the PTAP and YPXnL domains and the blocked PTAP-Tsg101 or YPXnL-Alix interactions both affected HIV-1 release [43]. We observed that both the PLPI and YGPL motifs of BGag played an essential role during virus budding. The inhibition of virus release was more obvious when two L domain motifs were mutated simultaneously. In the present study, immunoprecipitation verified that the interaction between BGag with mutations of the two L domains and Alix or Tsg101 was weakened to a certain extent, which proved that the L domains are important for BFV recruitment of ESCRT. The relevant mechanism and the corresponding relationship between the two L domains (PLPI and YGPL) and Alix or Tsg101 require further investigation. In addition, we found that V498 of Alix and N69 of Tsg101 were important for BFV VLP budding. We confirmed the interaction between V498D of Alix or N69P of Tsg101 with BGag. As shown in Figure S3, the interaction between BGag and V498D was weakened significantly compared with that of wild-type Alix. This might be the reason why V498D could not effectively rescued the inhibition of VLP budding by *ALIX* knockdown (Figure 5B). As shown in Figure S4, interestingly, the results showed that the interaction between BGag and N69P was significantly enhanced compared with that in wild-type Tsg101. Combined with the results in Figure 6B, N69P could not compensated for the reduction in BFV VLP budding caused by *TSG101* knockdown, which was not caused by the change of interaction between N69P and BGag. This might reflect the fact that N69P cannot efficiently recruit ESCRT complexes despite its strong interaction with BGag, which needs to be verified using further experiments.

To date, three consensus sequences of L domains have been characterized, a P(T/S)AP L domain motif, a PPXY L domain motif, and a YPXL L domain motif. In this study, we found that PLPI and YGPL in BGag were novel motifs that differ from the previously discovered motifs. Furthermore, the PLPI motif and the YGPL motif have high homology to the prototypic PPXY and YPXL L domains. Interestingly, the YXXL motif is conserved in all FV isolates from different species [5]. There are two YXXL motifs in BGag, YGPL and YAIL, respectively (Figure 2A). In this study, mutations in the YGPL motif resulted in an approximately five-fold reduction in BFV particle release, and analysis using motif interchange showed that YGPL is a motif with L domain function (Figure 3). Mutations of the YAIL amino acids in BGag had no effect on particle release, suggesting that it has no classical L domain function (Figure 2). Previous studies have shown that the YEIL in PFV Gag has no classical L domain function but was important for capsid assembly [27]. Further comparison showed that both the YAIL motif in BGag and the YEIL motif in PGag were YXXLGL-like motifs. In addition, the YGPL L domain motif in BGag belongs to the YXXL-like motif group, but not the YXXLGL-like motif group. YEILGL is important for the correct capsid assembly of PFV [44]; therefore, the influence of YAILGL on BFV capsid assembly deserves further investigation. Overall, these results suggested that the conserved YXXL motif in FV Gag might plays an important role in certain steps of the FV life cycle.

Like other retroviruses, the Gag protein of BFV interacts with the intracellular trafficking machinery in a complex manner, which is consistent with its multiple functions in viral replication. Studies have shown that the Gag proteins of several retroviruses shuttle between the nucleus and cytoplasm, including those of murine leukemia virus (MLV), PFV, RSV, HIV, and mouse mammary tumor virus (MMTV) [45,46,47,48,49]. PFV Gag has been reported to be trafficked into the nucleus twice during its life cycle, including early-stage entry into the nucleus as part of the pre-integration complex (PIC) and trafficking transiently through the nucleus during virus particle assembly in the late stage [38]. However, the cellular transport pathways and molecular mechanisms required for the newly synthesized Gag to travel between the nucleus and cytoplasm remain unclear. Our results showed that mutations in PLPI and YGPL led to the retention of BGag in the nucleus, which was detrimental to the efficient assembly of the virus. Considering that BGag interacted with Alix and Tsg101 through the two L domains, it would be interesting to follow up on the relationship between these interactions and the ability of BGag to shuttle between the nucleus and cytosol, which is currently unclear.

In summary, we first confirmed that there were L domains in BGag that interact with Alix and Tsg101, which recruit the ESCRT pathway to mediate virus budding. PLPI and YGPL are the two L domain motifs of BFV, which differ from the classical L domain. These results suggest that some viruses without the classical L domain motifs in their structural proteins might also use ESCRT to promote budding. Hence, our findings provide new insights into the viral proteins that recruit ESCRT components, thus advancing the study of the molecular mechanisms of virus budding.

## Data Availability

The data presented in this study can be provided upon request from the corresponding author.

## Supplementary Materials

The following supporting information can be downloaded at: https://www.mdpi.com/article/10.3390/v14030522/s1, Figure S1: (A) High similarity of ESCRT proteins between human and bovine. The same number of HEK293T or MDBK cells (1 × 106) were lysed with lysis buffer. The corresponding proteins were measured by western blot. The primary antibodies were rabbit anti-homo Alix, rabbit anti-homo Vps4 or mouse anti-homo Tsg101. (B) MDBK (2 × 106) cells were transfected with 3HA-BEnv and either the wild-type or L2/L3 mutated BGag and harvested at day 2 post-transfection. The cell culture supernatants were filtered with a 0.45 µm filter and purified by ultracentrifugation. Transfected cells were lysed using lysis buffer. Levels of proteins in cells and supernatants were measured using western blot. VLPs release levels were quantified according to the method described in Statistical Analysis; Figure S2: Quantification of nuclear and cytosol distribution of wild-type or L2/L3 mutanted BGag proteins. The amount of BGag in nuclear or cytosol were normalized against the amount of their respective Histone or GAPDH loading control. Corresponding band intensities were determined by Image J. The data are the averages of three independent experiments. Compared with the wild-type (wt), * *p* < 0.05, ** *p* < 0.01, *** *p* < 0.001, **** *p* < 0.0001; Figure S3: Immunoprecipitation with HA antibody of HEK293T (4 × 106) cells cotransfected with eukaryotic expression plasmids encoding 3HA-Alix or 3HA-Alix V498D and 3Flag-BGag; Figure S4: Immunoprecipitation with HA antibody of HEK293T (4 × 106) cells cotransfected with eukaryotic expression plasmids encoding 3HA-Tsg101 or 3HA-Tsg101 N69P and 3Flag-BGag.
