# Supplementary material for: Characterization of Bovine Foamy Virus Gag Late Assembly Domain Motifs and Their Role in Recruiting ESCRT for Budding
Source: Viruses. 2022 Mar 3;14(3):522. doi: 10.3390/v14030522 (PMC8952818; doi:10.3390/v14030522)
Supplement: Supplementary file 1 [file viruses-14-00522-s001.zip › viruses-1613984-supplementary.pdf]

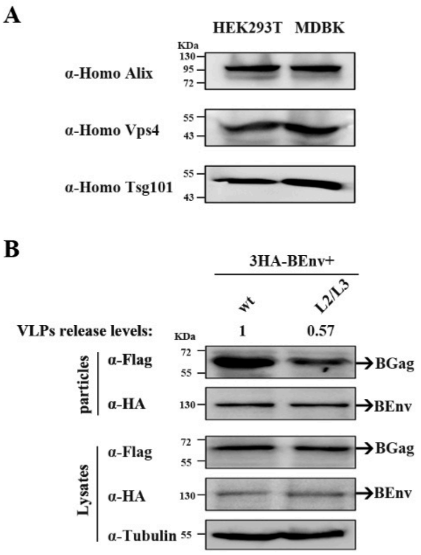

Supplementary Figure S1. (A) High similarity of ESCRT proteins between human and bovine. The same number of HEK293T or MDBK cells (1 × 10^6) were lysed with lysis buffer. The corresponding proteins were measured by western blot. The primary antibodies were rabbit anti-homo Alix, rabbit anti-homo Vps4 or mouse anti-homo Tsg101. (B) MDBK (2 × 10^6) cells were transfected with 3HA-BEnv and either the wild-type or L2/L3 mutated BGag and harvested at day 2 post-transfection. The cell culture supernatants were filtered with a 0.45 μm filter and purified by ultracentrifugation. Transfected cells were lysed using lysis buffer. Levels of proteins in cells and supernatants were measured using western blot. VLPs release levels were quantified according to the method described in Statistical Analysis.

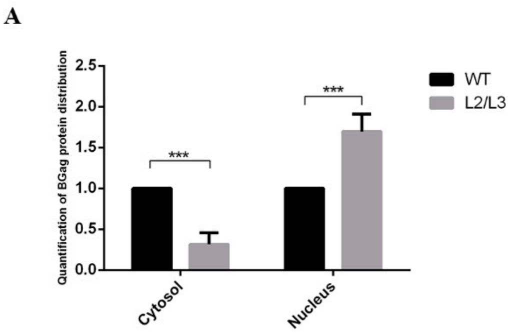

Supplementary Figure S2. Quantification of nuclear and cytosol distribution of wild-type or L2/L3 mutanted BGag proteins. The amount of BGag in nuclear or cytosol were normalized against the amount of their respective Histone or GAPDH loading control. Corresponding band intensities were determined by Image J. The data are the averages of three independent experiments. Compared with the wild-type (wt), * $p < 0.05$, ** $p < 0.01$, *** $p < 0.001$, **** $p < 0.0001$.

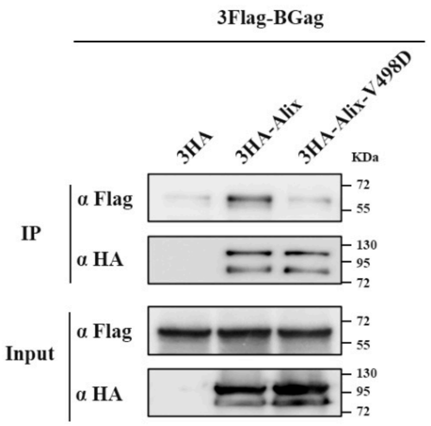

Supplementary Figure S3. Immunoprecipitation with HA antibody of HEK293T (4 × 10^6) cells cotransfected with eukaryotic expression plasmids encoding 3HA-Alix or 3HA-Alix V498D and 3Flag-BGag.

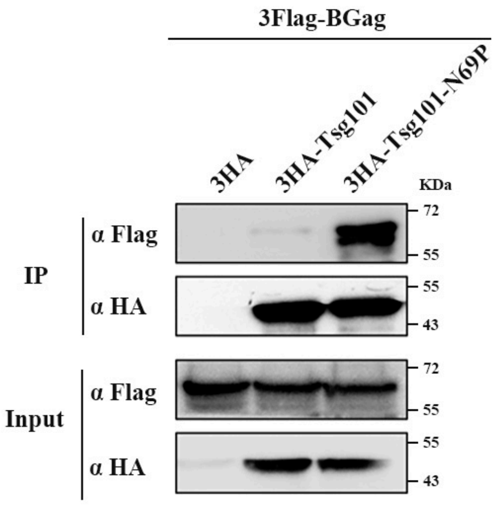

Supplementary Figure S4. Immunoprecipitation with HA antibody of HEK293T (4 × 10^6) cells cotransfected with eukaryotic expression plasmids encoding 3HA-Tsg101 or 3HA-Tsg101 N69P and 3Flag-BGag.
